# Identification of violence in the hospital environment: perceptions of nurses

**DOI:** 10.1192/j.eurpsy.2022.1796

**Published:** 2022-09-01

**Authors:** M. Mnif, N. Smaoui, R. Feki, I. Gassara, S. Omri, N. Charfi, J. Ben Thabet, L. Zouari, M. Maalej, M. Maalej

**Affiliations:** Hedi Chaker University Hospital, Psychiatry C, Sfax, Tunisia

**Keywords:** violence, psychiatric departments, emergency departments

## Abstract

**Introduction:**

Violence is recognised by the WHO as a major and ubiquitous public health problem, that is constantly worsening.

**Objectives:**

The aim of our work was to estimate the frequency of aggressions against nurses in psychiatric and emergency departments, and to identify the factors associated with it.

**Methods:**

This was a cross-sectional, descriptive and analytical study. It took place between January and March 2021, at both hospitals of Sfax (Tunisia).This study targeted 60 nurses in the psychiatry and emergency services.

**Results:**

The sample comprises 35 nurses (58%) from psychiatric services and 25 nurses (42%) from emergency services, mainly females (51%) and with average age of 35 years.(+/-9). The nurses interviewed were exposed to violence quite often, in 93% of cases. Almost all nurses (90 %) experienced verbal violence, 70 % experienced physical violence and more than half (62 %) experienced psychological violence. Nearly 11% of nurses reported a sexual violence. Factors that explain or contribute to violence mentioned by the participants were as follows; The Verbal violence was significantly correlated with poor reception conditions (p=0.013). The Sexual violence was significantly correlated with young age of nurse (p=0.005). As for psychological violence, it was significantly correlated with work overload (p=0.004), a poor caregiver-patient relationship (p=0.02) and poor patient care (p=0.04).

**Conclusions:**

Our study showed that violence against nurses was frequent in psychiatric and emergency departments. Various factors could modulate their occurrence such as training and improvement of the working conditions.

**Disclosure:**

No significant relationships.

